# The Structural Understanding of Transthyretin Misfolding and the Inspired Drug Approaches for the Treatment of Heart Failure Associated With Transthyretin Amyloidosis

**DOI:** 10.3389/fphar.2021.628184

**Published:** 2021-02-18

**Authors:** Shan He, Xinyue He, Lei Liu, Wenbo Zhang, Lanlan Yu, Zhun Deng, Zhang Feiyi, Shanshan Mo, Yue Fan, Xinyue Zhao, Lun Wang, Chenxuan Wang, Shuyang Zhang

**Affiliations:** ^1^ Department of Cardiology, Peking Union Medical College Hospital, Peking Union Medical College and Chinese Academy of Medical Sciences, Beijing, China; ^2^ Department of Chemistry, University of Wisconsin-Madison, Madison, WI, United States; ^3^ State Key Laboratory of Medical Molecular Biology, Institute of Basic Medical Sciences, Chinese Academy of Medical Sciences and Peking Union Medical College, Beijing, China; ^4^ Institute for Advanced Materials, Jiangsu University, Zhenjiang, China

**Keywords:** Transthyretin, amyloidal aggregation, protein structural stabilizers, cardiac amyloidosis, protein folding

## Abstract

Substantial controversies exist in the exploration of the molecular mechanism of heart failure (HF) and pose challenges to the diagnosis of HF and the discovery of specific drugs for the treatment. Recently, cardiac transthyretin (TTR) amyloidosis is becoming recognized as one of major causes of underdiagnosed HF. The investigation and modulation of TTR misfolding and amyloidal aggregation open up a new revenue to reveal the molecular mechanisms of HF and provide new possibilities for the treatment of HF. The aim of this review is to briefly introduce the recent advances in the study of TTR native and misfolding structures, discuss the correlation between the genotype and phenotype of cardiac TTR amyloidosis, and summarize the therapeutic applications of TTR structural stabilizers in the treatment of TTR amyloidosis-associated HF.

## Introduction

Heart failure (HF) is a clinical syndrome that originates from the insufficient cardiac output to accommodate the need of peripheral tissues or organs due to the cardiac systolic or diastolic dysfunction (McMurray and Pfeffer, 2005). HF is a global pandemic, affecting at least 40 million people worldwide. Its prevalence increases with aging populations (Triposkiadis et al., 2019). Roughly 2% of adults suffered from this disease and this ratio further increases by 4–8% for the senior population with ages of 65 years or older (GBD 2015 Disease and Injury Incidence and Prevalence Collaborators, 2016). Nowadays, HF is emerging as one of leading causes of hospitalization among elderly people. The treatment of symptomatic HF is a notorious challenge with a 5-years survival rate of 50% (Metra and Teerlink, 2017). HF has imposed a huge economic burden to global healthcare systems. Globally, about $108 billion must be spent annually for the treatment of HF including $65 billion in direct medical costs and $43 billion attributed to indirect costs (GBD 2015 Disease and Injury Incidence and Prevalence Collaborators, 2016). Due to the high prevalence, poor prognosis, and enormous burden on healthcare systems, it raises an urgent requirement for a better understanding of the mechanism of HF. There are many factors that cause HF including cardiac factors (e.g., coronary artery disease, heart attack, hypertension, faulty heart valves, cardiomyopathy, myocarditis, congenital heart defects, and heart arrhythmias), and non-cardiac factors (such as diabetes, emphysema, HIV, hyperthyroidism, hypothyroidism, hemochromatosis, severe forms of anemia, amyloidosis, drug or alcohol misuse, and certain medications). The treatment for HF include pharmacologic treatments (such as the administration of angiotensin-converting enzyme inhibitor (or angiotensin II receptor blockers) and beta blocker), aerobic exercise, and symptom treatment (such as diuretic, sodium restriction, digoxin). The mechanism research providing valuable targets for drug discovery can be translated to improve the treatment of HF. However, many pathogenic mechanisms leading to HF have been proposed, such as neurohormonal activation, inflammation, abnormal immunity activation, amyloidogenesis, increased hemodynamic overload, ischemia-related dysfunction, which cause the comprehension in the clinical treatment of HF. Substantial challenges and debates remain in the exploration of the molecular mechanism of HF.

Currently, extensive attempts have been made toward understanding the mechanisms that cause the development of HF. Distinct hypotheses have been made to explain the occurrence of HF from different perspectives including neurohormonal activation, inflammation, and immunity (Zhang et al., 2017; Lam et al., 2018; Stanciu, 2019). 1) Neurohormonal activation (Lam et al., 2018). This theory hypothesizes that when the cardiac output is greatly reduced at the early stage of HF, the activation of the adrenergic nervous system and the renin-angiotensin-aldosterone system (RAAS) generates compensatory feedbacks (strengthened cardiac contractility, peripheral vasoconstriction, and sodium and fluid retention) to maintain the effective circulating volume. The consequences of neurohormonal activation are two-sided: a sufficient circulation in a short-term period and a cardiac remodeling as a long-term negative outcome. Increased plasma levels of several neurohormonal biomarkers (e.g., plasma renin, aldosterone, norepinephrine, and endothelin 1) promote fibroblast proliferation, extracellular matrix deposition, and increased oxidative stress. Thus, it leads to a series of decompensated cardiac dysfunctions, such as cardiac fibrosis, dilated chamber, weakened contractile function, and decreased stroke volume (Lam et al., 2018). 2) Inflammation (Stanciu, 2019). Pro-inflammatory cytokines, including interleukin-1β (IL-1β), interleukin-6 (IL-6), and tumor necrosis factor-α (TNF-α) are produced by the stimulated cardiomyocytes. Pro-inflammatory cytokines promote the expansion of macrophages in the cardiac tissues and the recruitment of peripheral blood monocytes to heart. As a result, the inflammatory cascade is magnified and triggers cardiac hypertrophy and cardiac fibrosis (Stanciu, 2019). 3) Immunity (Zhang et al., 2017). In the state of heart damage, dendritic cells (DCs) are switched to a mature state with enhanced antigen-presenting capacity and facilitate the release of pro-inflammatory cytokines. Subsequently, mature DCs induce the differentiation of native T cells into CD4^+^ T cells and CD8^+^ T cells, leading to a breaking self-tolerance, i.e., the cytotoxicity and adaptive immune response to heart tissue (Zhang et al., 2017).

**FIGURE 1:**
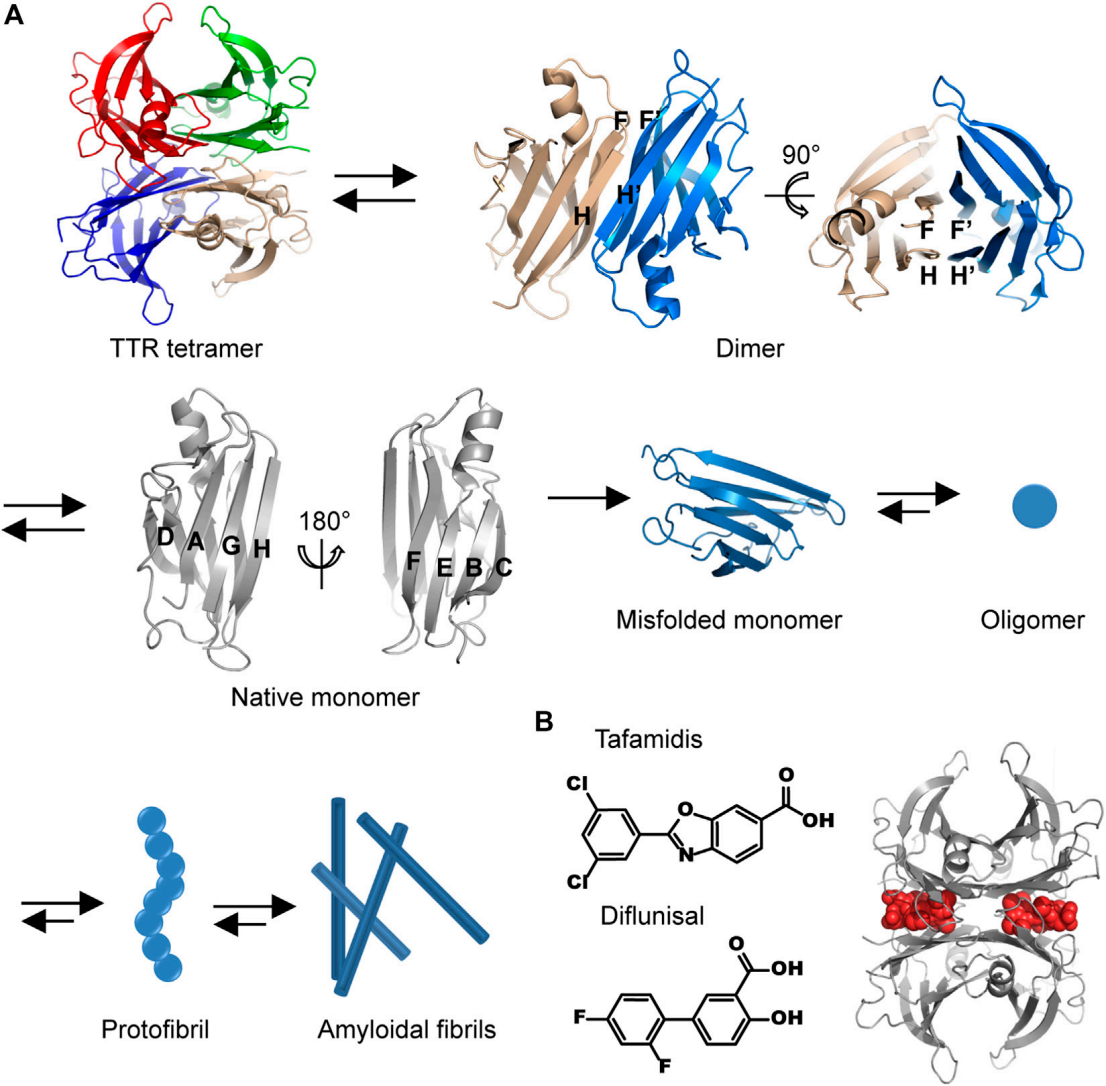
The molecular pathway of TTR amyloidal aggregation. **(A)** The structural illustration of the dissociation of TTR tetramer into monomer and the aggregation of TTR misfolded monomer. Different protein chains are indicated by different colors. The structures of TTR tetramer, dimer, and monomer are drawn by using the crystallographic data of PDB ID 1F41. **(B)** The chemical structures of tafamidis and diflunisal **(left)** and the representative model of structural stabilizers occupying the thyroxine binding site (right, the structure is drawn by using the crystallographic data of PDB ID 2ROX). Gray, a TTR tetramer; red, two binding structural stabilizers.

The mechanisms discussed above have been applied to guide the therapeutic pharmacy design. However, the discovered drugs are unsuccessful in the treatment of the HF patients with characteristic amyloid deposits in myocardium. A recent investigation of cardiac amyloidosis prevalence revealed that the incidence rate of cardiac amyloidosis among the hospitalized patients in the United States increased substantially since 2000 from 18 per 100,000 person-years to 55 per 100,000 person-years in 2012 (Gilstrap et al., 2019). The mainly amyloidal components that infiltrate in the heart causing cardiac amyloidosis are immunoglobulin light chain amyloid fibril protein (AL) and transthyretin amyloidosis (ATTR), and thus cardiac amyloidosis can be classified into AL-cardiac amyloidosis (AL-CA) and TTR-cardiac amyloidosis (ATTR-CA) (Benson et al., 2018). Emerging results suggest that AL-CA is responsible for 70% of patients with cardiac amyloidosis (Oerlemans et al., 2019). The population prevalence of ATTR-CA is less certain, may be responsible for 30% of HF patients with preserved ejection fraction aged 75 or older (Maurer et al., 2017; Oerlemans et al., 2019). TTR is mainly synthesized in the liver and the brain choroid plexus, circulating in peripheral plasma and cerebrospinal fluid (Fleming et al., 2009). The main physiological activity exerted by TTR is thought to be a transporter of thyroxine and retinol. TTR may also participate in the maintenance of normal cognitive processes and nerve regeneration during ageing (Fleming et al., 2009). As shown in Figure 1A, pathological TTR amyloidosis is a multiple-step protein assembly process involving: 1) amino acid mutations lead to a dissociation of native TTR tetramers into abnormally TTR monomers; 2) misfolded TTR monomers associate into oligomers and protofibrils; 3) TTR oligomers and protofibrils elongate to form mature amyloidal fibrils (Dobson, 2003). The exposure of TTR amyloidal intermediates, i.e., oligomers and protofibrils, to myocardial cells induces caspase-3 activation, triggering the onset of programmed cell death (Sousa et al., 2001). It leads to the degradation of myocardial cells, cardiac atrophy, and progressive infiltration, in which the receptors of advanced glycosylation end products (RAGE) also participate the organ injury generated by ATTR. Consequently, the deposition of ATTR results in cardiac conduction abnormalities and diastolic dysfunction (Marcoux et al., 2015; Michels da Silva et al., 2019). The prevalence of TTR amyloid deposit in heart is estimated to be 13% in the HF patients aged 60 or older with preserved ejection fraction. This proportion further increases to 32% for the patients aged 75 and older (González-López et al., 2015). The average age of onset for wild-type ATTR is 75 years and for hereditary ATTR is 45 years (Nakagawa et al., 2016; He et al., 2019). Cardiac ATTR is frequently accompanied by debilitating neurological with or without cardiac complications (Traynor et al., 2019). For the HF patients aged 60 or older with ATTR, they may also suffer from hypertension, coronary heart disease, and chronic obstructive pulmonary disease, etc. The amyloidosis of TTR is regarded as an underdiagnosed cause of HF and provides a new avenue to understand the mechanism of HF development.

In this review, we summarize the recent structural and medical advances of cardiac TTR amyloidosis studies. We illuminate the potential relationship between the genotype and phenotype of ATTR, discuss the impact of amino acid mutation on TTR activities, and outline the current therapeutic approaches against ATTR in clinical practice.

## The Structural Conversion of TTR Amyloidogenesis

Elucidating the TTR conformational change from native state to misfolded state is crucial for uncovering the molecular mechanism of TTR amyloid fibrosis. Substantial technical efforts have been applied toward studying the structural conversion of TTR using a series of experimental approaches, such as X-ray crystallography, nuclear magnetic resonance (NMR) spectroscopy, and cryo-electron microscopy (Cryo-EM), despite the heterogeneity and polymorphism in TTR folding and assembly manner, which create a challenge for structural characterization.

X-ray crystal diffraction experiments revealed that the native human TTR is folded into a globular structure consisting of eight antiparallel strands (denoted as from A to H) and one short α-helix (Wojtczak et al., 2010; Palaninathan, 2012) (Figure 1A). The eight *ß*-strands are associated into two groups of twisted *ß*-sheets. Specifically, A, D, G, and H strands are tethered by interstrand hydrogen bonds into an inner *ß*-sheet, whereas B, C, E, and F strands constitute an outer *ß*-sheet. The inner and outer *ß*-sheets are packed with each other in a layer-by-layer manner. The H and F strands are located at the edges of *ß*-sheets, providing active sites for interacting with the H′ and F′ strands from the adjacent TTR monomer, respectively. Thus, two TTR monomers are held together to form a dimer *via* interstrand hydrogen bonds. The dimerization of TTR renders two inner *ß*-sheets to form an extended *ß*-sheet pleat containing of A, D, G, H, and A′, D′, G′, H’ strands. Two inner extended *ß*-sheet pleats further wrap around each other, generating a dimer-dimer interface. Consequently, native human TTR is organized as a tetrameric cluster possessing an inner core (A, D, G, and H strands) and presenting an outer surface (B, C, E, and F strands).

*In vitro* experiments revealed that acidic pH conditions and the mutations identified from patients induce the dissociation of TTR tetramer into monomers and trigger the misfolding of monomer into amyloidal aggregates (Vieira and Saraiva, 2014). The destabilization of TTR tetrameric structure can be tracked by NMR. The acidification of solution triggers the flexible loop region between A and B to interact with A strand, leading to conformational changes occurring in the inner core domain (Lim et al., 2016). The impact of single amino acid mutation on the stability of native TTR tetramer is attributed to a propagation of structural perturbation from the mutation site to the entire inner/outer *ß*-sheet domains (Leach et al., 2018). The transmission electron micrographs showed that the TTR amyloid aggregates are rigid and unbranched fibrils with several micrometers in length and 70 to 130 Å in diameter (Serpell et al., 1995). A TTR fibril composes of two to three protofilaments. Each protofilaments are 25–35 Å in diameter and interwind with each other (Serpell, 1996). Synchrotron X-ray fiber diffraction pattern of TTR aggregate showed an intense 4.7 to 4.8 Å reflection on the meridian (Serpell, 1996; Sunde et al., 1997). It demonstrates that the periodicity of TTR protein arrangement along the fibril axis is 4.7–4.8 Å. This result suggests that the misfolded TTR proteins adopt a typical cross-β conformation and are stacked perpendicular to the elongation of protofilament (Serpell, 1996; Sunde et al., 1997). Multiple complementary methods have been used to reveal the atomic level structural model for the misfolded TTR aggregate, including scanning tunneling microscopy (STM) and X-ray crystallography for the key segments of TTR, and Cryo-EM for the full length of TTR. By using STM, the assembly structures of TTR 105–115 segment were observed by Yu et al. at a single-molecular level (Yu et al., 2018). In the STM images, TTR 105–115 segments present as linear lamella structures. Individual peptides are parallel to each other and packed into a protofilament-like structure. The distance between two adjacent peptide axes is 4.0 ± 0.2 Å, which is consistent with the structural features of cross-β conformation. Saelices et al. analyzed the crystal structures of 13 hexapeptide segments derived from the key amyloidogenic domains of TTR and found distinct assembly behaviors encoded by different TTR regions (Saelices et al., 2018). The segments from the C-terminal region of TTR (corresponding to the residues from 50 to 127) were observed to form in-register *ß*-sheets and pair as steric zippers. For instance, the crystalline structure of TTR 80–85 segment (80KALGIS85) shows that the TTR 80–85 *ß*-strands are assembled into anti-parallel *ß*-sheets. The side chains protruding from the surface of *ß*-sheet drive two *ß*-sheets to stack in layer-by-layer and form a tightly interdigitating steric zipper interface that is free from water. In contrast, the segments from the N-terminus (corresponding to the residues from 1 to 50) form out-of-register *ß*-sheets. For example, the anti-parallel *ß*-sheet formed by TTR 28–33 segment (28VAVHVF33) exhibits a wet and loosely stacking interface to interact with another TTR 28–33 *ß*-sheet, whereas the side chains from the same *ß*-sheet are self-complementing. To understand the folding structure of a full length TTR protein, Schmidt et al. investigated the molecular arrangement in a TTR fibril extracted from the tissue of a patient with hereditary Val30Met ATTR by Cryo-EM (Matthias Schmidt et al., 2019). The Cryo-EM experiment revealed that the misfolded TTR Val30Met protein consists of three *ß*-arch regions (TTR 11–35, 70–111, and 106–123) and 13 *ß*-strands (i.e., TTR 12–16, 19–21, 25–34, 60–62, 64–66, 70–73, 75–77, 79–80, 91–99, 103–104, 107–108, 114–115, and 118–122). Consistent with the X-ray crystallography results from TTR segments, the C-terminal motif of full length TTR Val30Met Cryo-EM structure is folded into multiple in-register *ß*-sheets. Whereas the N-terminal motif of TTR Cryo-EM structure presents one out-of-register *ß*-hairpin region involving the residues from 11 to 35 and two unstructured regions including the regions 1–10 and 36–50. Different from the key segment crystal structures, the full length TTR adopts a parallel *ß*-sheet conformation in the Cryo-EM structure.

## TTR Genotype and Cardiac Phenotype

ATTR is relatively common in the men older than 60 years and often regarded as senile amyloidosis (Grogan et al., 2016). Any step during the conversion from the soluble native state to amyloidal fibrils may significantly alter the progression of fibril formation, such as acidification, proteolysis, and the co-assembly with other non-fibrous substances. For example, the co-existence of glycosaminoglycan and serum amyloid *P* component obviously accelerates the misfolding and association of TTR (Kisilevsky, 2000; Zhang and Li, 2010). Point mutation in the TTR gene is another key factor that causes the occurrence of TTR-associated HF. Hereditary transthyretin amyloid cardiomyopathy (ATTR-CM) is an autosomal dominant single-gene genetic disease (Damrauer et al., 2019). Up to now, more than 120 types of point mutations have been identified (Ruberg et al., 2019). The common symptoms of hereditary ATTR (hATTR) typically include progressive infiltration of amyloidal deposit, early right-sided heart failure, and late systolic ejection fraction reduction. Patients could also have syndromes of peripheral neuropathy, autonomic neuropathy, cardiomyopathy, eye disease, and spinal amyloidosis (Koga et al., 2003; Rapezzi et al., 2010; Maurer et al., 2017). Changes in the heart function caused by TTR amyloidal deposition include cardiac systolic or diastolic dysfunction. Patients usually suffer from orthostatic hypotension and cannot tolerate RAAS inhibitor treatment. Some severe cases could result in HF and suffer from syncope caused by arrhythmia or heart block, and angina or infarction caused by amyloid deposits in the coronary arteries (Finsterer et al., 2018). In addition to heart diseases, other illnesses generated by hereditary TTR include polyneuropathy, sensorimotor polyneuropathy, glaucoma, intravitreal deposition, nephrotic syndrome, and renal failure (Michels da Silva et al., 2019). General diagnosis of hereditary TTR requires extracardiac tissue biopsy (lower fat, small salivary glands, rectal mucosa or kidney, and nerve) (Ando et al., 2013; Jamet et al., 2015). The TTR amyloidal deposit appears as an amorphous transparent substance in the optical microscopic image and can be labeled by Congo red or thioflavin T immunohistology (Schönland et al., 2012). The diagnosis of ATTR-CM requires an intracardiac tissue biopsy to manifest the presence of TTR amyloid deposits in the myocardium (Ruberg et al., 2012). In the clinical examination, a low electrocardiography (ECG) voltage in which the amplitudes of all the QRS complexes in the limb are smaller than 0.5 mV in the limb is frequently observed (Damy et al., 2016). Some patients may also exhibit one or more of the following diagnostic characteristics: a speckled appearance of the myocardium on echocardiography with end-diastolic thickness of the ventricular septum >1.2 cm, excessive uptake of technetium-99 m (Tc-99m) scintillation agent by the heart with ATTR amyloidosis, delayed enhancement of subendothelial gadolinium on cardiac magnetic resonance imaging (MRI), multiple occurrences of osteolytic lesions of myeloma, and cystic bone lesions of dialysis-related amyloidosis (Sousa et al., 2001; Jurcut et al., 2010). It is necessary to point out that imaging techniques only provide supporting evidences, and the gold standard for the diagnosis of ATTR is still lacking. The current diagnosis in clinical practice for ATTR-CM includes: 1) a noninvasive nuclear scintigraphy by using planar and SPECT imaging; 2) an invasive endomyocardial biopsy, i.e., the histology with Congo red staining with apple-green birefringence; and 3) a genetic testing to determine if the disease is due to a hereditary mutation in the *TTR* gene (Gillmore et al., 2016; Maurer et al., 2018; Dorbala et al., 2019).

Recorded TTR mutations associated with cardiac phenotypes includes Val122Ile, Thr60Ala, Leu111Met, Ile68Leu, Val30Met, Val30Met, Phe33Leu, Asp38Val, Asp38Val, Gly47Glu, Gly53Glu, Glu54Lys, Leu55Pro, Glu61Lys, Tyr69His, Ser77Tyr, His88Arg, and Tyr114Cys (Maurer et al., 2016; Damy et al., 2019). Point mutations mostly locate at the terminal regions of the TTR protein, resulting in an abnormal folding structure and diminished tetramer stability. TTR mutation sites are diverse and could lead to a variety of clinical symptoms. Familial amyloid polyneuropathy (FAP), familial amyloid cardiomyopathy (FAC), and familial leptomeningeal amyloidosis are the three main phenotypes of inherited ATTR amyloidosis. The main phenotype of heart disease in hATTR patients is aggressive cardiomyopathy (Sun et al., 2018). Val122Ile, Val30Met, Thr60Ala, Leu111Met, Ile68Leu showed obvious cardiac phenotypes in the large sample cohort (Reilly et al., 1995; de Carvalho et al., 2000; Sattianayagam et al., 2012; Quarta et al., 2015). The clinical manifestations are more frequent in middle-age and older men with severely damaged cardiac structure. Examined by echocardiography, invasive cardiomyopathy where strong echoes occur in the left ventricular myocardium is accompanied by pericardial effusion and atrial enlargement (González-López et al., 2017). Val122Ile is the frequently occurred mutation that causes FAC (Quarta et al., 2015). Patients with congestive heart failure, conduction block, and intractable arrhythmia need the implantation of pacemakers with/without cardioverter defibrillators (Maurer et al., 2016). Val30Met mutation usually causes FAP. Cardiac manifestations are mainly manifested in different types of heart block, and most patients require pacemaker implantation (Damy et al., 2019). Patients with Thr60Ala mutation can develop late-onset restrictive cardiomyopathy as well as sensorimotor and autonomic polyneuropathy (Zanazzi et al., 2019). Patients with Leu111Met mutation have a higher propensity to develop carpal tunnel syndrome as the initial manifestation and a liver transplantation is necessary for the treatment (Liepnieks and Benson, 2007). In a family study of patients with Ile68Leu mutation, the presence of amyloid aggregates was observed in the spinal canal of patients (Salvi et al., 2003). Despite the heterogeneity of mutation sites, the clinical characteristics and natural history of cardiac phenotype also generate difficulty for the diagnosis and treatment of ATTR. A long-term follow-up study of the Asian population (23 patients) revealed that the overall survival rates at 12, 24, 36, 48, and 60 months after diagnosis were 77.8, 55.6, 38.9, 27.8, and 11.1%, respectively (He et al., 2019). The low prognosis of ATTR is correlated with two facts. First, the onset symptoms were not obvious in the heart. Within the 23 patients, only five patients were observed to possess a low left ventricle ejection fraction (ejection fraction <50%). Second, the clinical characteristics of ATTR were heterogeneous, e.g., 78.3% of patients had abnormal electrocardiography and 56.5% of patients displayed pseudoinfarct pattern. This result highlights the complexity in the ATTR phenotypes and the difficulty to avoid misdiagnosis in practice (He et al., 2019).

Motivated by the different clinical phenotypes of the above-mentioned TTR mutants, a series of studies were approached to investigate the pathogenic mechanisms of TTR mutants. For example, Wright et al. used ^19^F-NMR technology to monitor in real time and found that amyloidal aggregation was caused by V122I mutation (Benjamin et al., 2018). The dissociation of TTR tetramer into monomer is revealed to be the rate-limiting step. Jiang et al. demonstrated that the mutation of V122I makes the Gibbs free energy of tetramer 3 times higher than that of the wild-type, and thus the energetic barrier of tetramer dissociation is significantly reduced by the mutation of V122I (Jiang et al., 2001). The dissociation of TTR tetramers is accelerated by the V122I mutant in favor of kinetics and thermodynamics. It is worth to note that, the observation with TTR V122I mutant that the single site mutation at the key site of TTR promotes the dissociation of TTR tetramer is probably a general mechanism existing in the misfolding process of other types of TTR pathogenic mutations. As a summary, the cardiac phenotype-related pathogenic site accelerates the rate-limiting step of dissociation of tetramers into monomers, resulting in massive formation of TTR amyloidal aggregates and the accumulation of the clinical phenotype of HF.

## The Clinical Practice of TTR Structural Stabilizer

Current therapeutic approaches toward the treatment of ATTR-CM focus on drug administration and surgical operation. Because 95% of plasma TTR is synthesized from liver, liver transplantation was firstly proved as the only potentially curative treatment for TTR amyloidosis in 1990s (Adams et al., 2019). The transplanted healthy liver produced normal and stable form of tetramers, which lowers the concentration of amyloidogenic monomers in blood and halts disease progress (Muchtar et al., 2017). However, the transplantation faces several challenges including the limited supply of livers and the long-term usage of immune suppressive therapy (Heymann and Tacke, 2016). Therefore, the majority of patients with ATTR need more affordable and accessible options. The structural stabilizer preventing the dissociation of TTR tetramer is a promising candidate. Several stabilizers have been approved by the Food and Drug Administration (FDA) for the treatment of ATTR-CM worldwide. Herein, we describe the pharmacological mechanism of stabilizers and summarize its clinical data.

Vigorous screening experiments and structure-based drug design studies have been carried out to identify TTR tetramers stabilizers at molecule level. More than 1,000 small aromatic molecules dynamically stabilizing TTR tetramers have been designed and synthesized (Gavrin et al., 2012). Most of these compounds were designed as a mimic of thyroxine, a natural hormone that specifically binds to the cavity of tetrameric TTR. These species share a similar chemical skeleton: two aromatic rings conjugated by a linker. The introduction of a polar group, halogen, or alkyl group into the aromatic ring facilitates the formation of electrostatic interactions and hydrophobic interactions between the thyroxine mimics and the binding pocket in the TTR protein (Gavrin et al., 2012). As manifested in the co-crystal structure of TTR binding with thyroxine mimic, the synthetic mimic occupies the thyroxine binding site and does not disturb TTR’s native structure (Gavrin et al., 2012). At low pH conditions, where TTR tetramers are prone to aggregate, the presence of thyroxine mimic in TTR solution greatly improve the stability of TTR against denaturation (Wiseman et al., 2005). The plausible mechanism underlying the stabilizing impact by thyroxine mimic is attributed to the elevated energy barrier of TTR tetramer dissociation leading to less amyloidal fibril formation and thus reducing the rate of amyloidogenesis progression (Johnson et al., 2012). As for increasing the bioavailability of compound, it is important to avoid the non-specific binding of thyroxine mimic to the abundant proteins in serum and increase the selectivity of the compounds toward thyroxine TTR binding site (Connelly et al., 2010).

Two TTR kinetic stabilizers, tafamidis and diflunisal, have been selected and used in clinical trials (chemical structures are shown in Figure 1B). Tafamidis, a non-steroidal anti-inflammatory drug (non-NSAID) benzoxazole derivative, is the first stabilizer approved to halt the progression of cardiac impairment in TTR familial amyloid cardiomyopathy (Maurer et al., 2018). The tafamidis phase III transthyretin amyloidosis cardiomyopathy (ATTR-ACT) trial is a multicenter blinded study in 441 patients with ATTR-CM who received placebo 20 mg, tafamidis 20 mg, or tafamidis 80 mg daily with a randomization ratio of 2:2:1 (Table 1). This investigation exhibits a striking cardiovascular outcome in the patients with oral intake of tafamidis at 80 mg/day (Maurer et al., 2018). Over a period of 30 months, all-cause mortality decreased from 42.9% to 29.5%, and cardiovascular-related hospitalizations reduced from 0.70 to 0.48 annually. Both of mortality and morbidity endpoints all achieved statistical significance. This remarkable result proves that 80 mg dose of tafamidis not only relieves symptoms of HF but also improves a long-term prognosis of ATTR-CM. The general molecular mechanism of structural stabilizer can be applied to interpret the pharmacological activity that tafamidis selectively occupies the thyroxine binding site, preventing the structural conversion from native tetramer to denatured monomers (Figure 1B). Consequently, the possibility of TTR misfolding and amyloidal aggregation is substantially reduced (Bulawa et al., 2012). Tafamidis circulates in serum or plasma mainly in the form of binding with plasma proteins (99%) and undergoes the metabolic pathway through glucuronidation (Bulawa et al., 2012). Up to date, Tafamidis has been approved as the first-line exclusive drug in the treatment of TTR amyloidosis in US, Europe, and Japan.

Diflunisal is a NSAID that was previously administered in the clinical practice as an antipyretic, analgesic, and anti-inflammatory agent (Enthoven et al., 2016). Recent investigations revealed that diflunisal can be applied to facilitate nervous and cardiac repair and regeneration (Miller et al., 2004; Sekijima et al., 2006). In a clinical trial including 120 patients with TTR cardiac amyloidosis demonstrates that an increased survival is associated with diflunisal treatment (Rosenblum et al., 2017) (Table 1). In an international randomized double-blind trial, a total of 130 patients received diflunisal or placebo twice daily for 2 years (diflunisal 250 mg, *n* = 64; placebo group, *n* = 66) (Berk et al., 2013) (Table 1). Compared to the placebo group, diflunisal significantly slowed down the progression of nervous amyloidal disease and enhanced the cardiac functions. Nowadays, diflunisal has been approved by FDA for the clinical application, but some side effects have also been reported. For example, diflunisal can lead to a decline (6%) in estimated glomerular filtration rate (Ikram et al., 2018). A chronical administration of diflunisal can slow down the renal blood flow and thus becomes inadvisable to some patients with renal insufficiency. Therefore, Diflunisal is a second line drug for ATTR-CM. In the future, randomized trials with large samples are needed to collect more evidence for evaluate the safety of diflunisal in the treatment of ATTR-CM.

## Conclusion

The investigations of TTR misfolding and amyloidal aggregation at molecular level have greatly improved the understanding the mechanism of HF and promoted the treatment of HF. The destabilization and dissociation of TTR tetramers into abnormally TTR monomers lead to the misfolded TTR monomers, which abnormally assemble into oligomers, protofibrils, and mature amyloidal fibrils. Structural stabilizers such as tafamidis and diflunisal can block the dissociation of TTR tetramer and facilitate the treatment of patients with TTR amyloidosis. With the gradual deepening of the research on the structure of TTR protein, there will be a profound understanding of the mechanism of TTR-associated HF. We note that, there still remain challenges in the research that need to be urgently studied, including: 1) the discovery of compounds to reverse the association state of TTR from amyloidal state to native folding state; 2) the misfolding structures of TTR amyloidal oligomers and the structure-based drug design to specifically target the amyloidal intermediates of TTR; 3) the therapeutic approaches to promote cardiac repair and preserve cardiac function against the dysfunction caused by TTR amyloidosis. The efforts made in these aspects will benefit the medical treatment of TTR-related patients in the future.

**TABLE 1:** Clinical trials of structural stabilizers in patients with ATTR-CM.

Trial	Design of trials	*N*	Treatment	Follow-up	Primary outcome
Tafamidis					
Maurer et al., 2018	International randomized, double-blind	441	Tafamidis 20 mg, or tafamidis 80 mg daily in a 2:2:1 randomization	30 months	All-cause mortality; cardiovascular-related hospitalizations
Diflunisal					
Rosenblum et al., 2017	Retrospective study	120	500 mg daily	16 years	The composite outcome of death or orthotropic heart transplant
			Non-randomization		
Berk et al., 2013	International randomized, double-blind	130	250 mg twice daily or placebo in a 1:1 randomization	2 years	Neuropathy impairment score plus 7 nerve tests
Castano et al., 2012	Retrospective study	13	250 mg twice daily non-randomization		Cardiac structure (left ventricular mass), function (ejection fraction)
